# Reusable Biosensor for Easy RNA Detection from Unfiltered Saliva

**DOI:** 10.3390/s25020360

**Published:** 2025-01-09

**Authors:** Paweł Wityk, Agata Terebieniec, Robert Nowak, Jacek Łubiński, Martyna Mroczyńska-Szeląg, Tomasz Wityk, Dorota Kostrzewa-Nowak

**Affiliations:** 1 Map Your DNA Ltd., Świerkowa 40, 83-330 Lniska, Poland; 2 Beckman Institute for Advanced Science and Technology, University of Illinois at Urbana-Champaign, Urbana, IL 61801, USA; 3 Department of Biopharmaceutics and Pharmacodynamics, Medical University of Gdańsk, Al. Gen. J. Halera 107, 80-416 Gdańsk, Poland; 4 Fungal Physiology, Westerdijk Fungal Biodiversity Institute and Fungal Molecular Physiology, Utrecht University, 3584 CS Utrecht, The Netherlands; terebieniecagata@gmail.com; 5 Institute of Physical Culture Sciences, University of Szczecin, 40B Piastów Al., Building 6, 71-065 Szczecin, Poland; robert.nowak@pum.edu.pl; 6 Department of Pathology, Pomeranian Medical University in Szczecin, 1 Unii Lubelskiej St., 71-242 Szczecin, Poland; 7 Faculty of Mechanical Engineering and Ship Technology, Gdańsk University of Technology, Narutowicza 11/12, 80-233 Gdańsk, Poland; jacek.lubinski@pg.edu.pl; 8 Faculty of Chemistry, Gdańsk University of Technology, Narutowicza 11/12, 80-233 Gdańsk, Poland; martyna.mroczynska@pg.edu.pl; 9 Department of Clinical and Molecular Biochemistry, Pomeranian Medical University in Szczecin, 72 Powstańców Wlkp. Al., 70-111 Szczecin, Poland; dorota.kostrzewa.nowak@pum.edu.pl

**Keywords:** biosensor, capacitance, infection, saliva, EIS

## Abstract

Biosensors are transforming point-of-care diagnostics by simplifying the detection process and enabling rapid, accurate testing. This study introduces a novel, reusable biosensor designed for direct viral RNA detection from unfiltered saliva, targeting SARS-CoV-2. Unlike conventional methods requiring filtration, our biosensor leverages a unique electrode design that prevents interference from saliva debris, allowing precise measurements. The biosensor is based on electrochemical principles, employing oligonucleotide probes immobilized on a hydrophobic-coated electrode, which prevents air bubbles and salt crystal formation. During validation, the biosensor demonstrated a sensitivity and specificity of 100%, accurately identifying SARS-CoV-2 in saliva samples without false positives or negatives. Cross-validation with RT-qPCR, the gold standard for COVID-19 diagnostics, confirmed the reliability of our device. The biosensor’s performance was tested on 60 participants, yielding 12 true positive results and 48 true negatives, aligning perfectly with RT-qPCR outcomes. This reusable, easy-to-use biosensor offers significant potential for point-of-care applications in various healthcare settings, providing a fast, efficient, and cost-effective method for detecting viral infections such as COVID-19. Its robust design, minimal sample preparation requirements, and multiple-use capability mark a significant advancement in biosensing technology.

## 1. Introduction

The COVID-19 pandemic [1] has emphasized the global need for rapid, accurate diagnostic tools, particularly for the detection of SARS-CoV-2, the causative virus. Early detection remains essential for controlling transmission, managing outbreaks, and implementing timely treatment [2,3,4]. However, the virus’s incubation period, which can range from 3 to 14 days, complicates early diagnosis based solely on clinical symptoms [5]. As a result, molecular diagnostic methods, including nucleic acid amplification tests (NAAT) like quantitative reverse transcription polymerase chain reaction, are the current gold standard for detecting the viral genome in patient samples [6].

Despite being widely adopted, RT-qPCR has several limitations [7]. While it can detect viral RNA with high sensitivity, the technique requires complex sample processing, including the isolation of viral RNA from biological fluids, for example, from nasopharyngeal swabs. This process is time-consuming, expensive, and requires specialized laboratory equipment and trained personnel. Additionally, variability in diagnostic sensitivity, which can range between 50% and 79%, may arise depending on the timing of sample collection and the methodology used. While serological tests can offer supplementary insights by detecting antibodies, their diagnostic value is restricted to the later stages of infection, typically after the immune response has developed. [8,9]

Saliva has gained attention as a promising alternative biological medium for SARS-CoV-2 diagnosing. It is non-invasive and easier to collect compared to nasopharyngeal swabs, making it more suitable for point-of-care (POC) diagnostics. Clinical studies have shown that saliva samples can reliably contain viral RNA, with reported sensitivities for qPCR tests as high as 90% when compared to nasopharyngeal samples. Despite its advantages, saliva presents a unique challenge for diagnostics due to its complex composition, which includes natural microflora, squamous cells, and various impurities. These components can inhibit nucleic acid amplification, necessitating filtration or other sample preparation steps to ensure reliable test results. Such additional procedures increase the complexity and cost of testing, making them less feasible for rapid diagnostics in resource-limited settings [10,11].

To overcome these challenges, this study presents a novel, reusable biosensor capable of directly detecting SARS-CoV-2 RNA from unfiltered saliva samples. Our innovative approach eliminates the need for pre-filtration by employing a unique cuvette design and advanced electrochemical detection methods. The biosensor is based on electrochemical impedance spectroscopy (EIS), a highly sensitive and versatile technique used to monitor changes in electrical properties at the electrode–sample interface, particularly in response to biomolecular interactions.

### 1.1. Impedance Spectroscopy in Biosensors

Impedance spectroscopy [12] is a well-established method for detecting biomolecular interactions, including the binding of nucleic acids, proteins, and other analytes. In biosensing, EIS measures the opposition (impedance) that the electrode surface presents to an alternating current. When a target molecule, such as viral RNA, binds to a biosensor’s electrode, it induces changes in the electrical properties of the interface. These changes are detected as variations in impedance, which can be correlated with the concentration of the target molecule [13,14].

In our biosensor, the electrode surface is modified with oligonucleotide probes designed to hybridize specifically with the SARS-CoV-2 RNA. The binding of viral RNA to these probes alters the impedance at the electrode surface, which is recorded and analyzed. EIS is particularly advantageous for this application because it provides real-time, label-free detection, allowing for direct measurement of the interaction between the probe and the viral RNA without the need for secondary reagents or complex amplification steps.

The biosensor is designed to operate with minimal sample preparation, making it suitable for point-of-care testing. The impedance measurements are highly sensitive to changes in the electrical properties of the sample, enabling the detection of viral RNA even at very low concentrations. In this study, we demonstrate the biosensor’s ability to detect RNA at concentrations as low as 1 aM, which is comparable to the sensitivity of RT-qPCR. This capability is critical for early-stage detection of viral infections, when viral loads may be low.

### 1.2. Cuvette Design for Enhanced Performance

A key feature of our biosensor system is the specially designed cuvette that houses the saliva sample and the electrode. Saliva [8,11] is a challenging medium for diagnostics due to its heterogeneity; it contains not only viral particles but also various impurities such as food particles, epithelial cells, and oral microbiota. These impurities can interfere with the biosensor’s measurements by obstructing the electrode surface or altering the impedance in unpredictable ways. To address this issue, we developed a cuvette with a downward-facing electrode configuration that leverages gravity to separate interfering particles from the sensing surface [15,16].

The cuvette design allows the saliva sample to be placed directly in the container, where the electrode surface is positioned at the bottom, facing downward. This orientation enables larger debris, epithelial cells, and other undesired particles to settle at the bottom of the cuvette, away from the active sensing surface. This minimizes interference from contaminants and ensures that the electrode remains exposed to the liquid phase of the sample, which contains the target viral RNA. Additionally, the cuvette is designed to hold a stable volume of saliva-buffer mixture, ensuring consistent and repeatable measurements [15,16].

The innovative electrode design also includes a protective hydrophobic coating, which prevents air bubbles and salt crystals from forming on the electrode surface during the measurement process. These features enhance the biosensor’s durability and allow for its reuse, making it a cost-effective alternative to single-use diagnostic tests. The ability to reuse the biosensor without significant loss of sensitivity or specificity is particularly important for high-demand settings, such as during a pandemic, where rapid and widespread testing is required.

### 1.3. Novelty of the Study

This study introduces a novel, reusable electrochemical biosensor that integrates impedance spectroscopy [17] with an innovative cuvette design for the detection of SARS-CoV-2 RNA from unfiltered saliva. By eliminating the need for sample pre-treatment, our biosensor significantly simplifies the diagnostic process, making it highly suitable for point-of-care applications. The combination of sensitive impedance measurements and an optimized cuvette design enables accurate and reliable detection of viral RNA even at low concentrations, offering a promising alternative to conventional RT-qPCR methods. With further validation, this technology could be adapted for the detection of a wide range of viral and bacterial infections, representing a significant advancement in biosensing for infectious disease diagnostics.

## 2. Materials and Methods

This study aimed to develop a reusable biosensor for detecting SARS-CoV-2 RNA directly from unfiltered saliva samples using electrochemical impedance spectroscopy (EIS). The biosensor was designed to overcome the limitations of traditional methods by eliminating the need for sample filtration or extensive preparation. Below, we detailed the materials, probe design, electrode preparation, functionalization process, and measurement procedures involved in this study.

### 2.1. Oligonucleotide Probe Design

The biosensor’s key detection element was a synthetic oligonucleotide probe designed to specifically target the SARS-CoV-2 RNA genome. The full genome sequences of SARS-CoV-2, along with related *Betacoronaviruses*, were retrieved from the National Center for Biotechnology Information (NCBI) and aligned using the MAFFT algorithm. This alignment identified unique regions within the SARS-CoV-2 genome that could serve as specific molecular targets. The selected target sequence for the probe was 5′-AGA TCA GTT TCA CCT AAA CTG TTC ATC A-3′, which exhibited minimal homology to other human or viral sequences, ensuring high specificity. The following probe was used: 5′-HS-TGA TGA ACA GTT TAG GTG AAA CTG ATC T-3′.

The oligonucleotide probe was synthesized with a 5′-thiol (-SH) group to facilitate covalent immobilization onto the gold electrode surface. The probe length was optimized to 20–28 nucleotides with a GC content of 35.7% to ensure stable binding and hybridization with the viral RNA. The melting temperature (T_m_) was set to 56.3 °C to ensure reliable performance under physiological conditions.

### 2.2. Electrode Preparation and Functionalization

The biosensor electrodes were gold-plated interdigitated electrodes (DropSens, Llanera, Spain) with bands and gaps of 5 µm. Each interdigitated electrode (IDE) features finger widths and spacings of 5 µm, with a total of 125 fingers. The combined length of the electrodes is approximately 14 cm, covering a surface area of around 10 mm^2^. Titanium is used as an adhesive layer. These electrodes were selected for their ability to form stable Au-S bonds with thiolated oligonucleotides, ensuring robust immobilization and durability for repeated use.

Prior to functionalization, the electrodes underwent a thorough cleaning process to remove organic and inorganic impurities. The cleaning solution consisted of 25% hydrogen peroxide (H_2_O_2_) and 50 mM sulfuric acid (H_2_SO_4_), which was applied to the electrode surface for 10–15 min. The electrodes were then extensively rinsed with ultrapure water to remove any residual cleaning solution.

Following the cleaning procedure, the oligonucleotide probes were immobilized onto the electrode surface. To prepare the probe (5′-HS-TGA TGA ACA GTT TAG GTG AAA CTG ATC T-3′) 100 µL of a 10 µM oligonucleotide solution was mixed with Tris(2-carboxyethyl)phosphine (TCEP)-agarose, then after 1 h centrifuged (4000× *g*, 15 min, 4 °C), to facilitate the reduction of disulfide bonds and ensure efficient attachment of the thiol group to the gold surface. The solution was incubated with the electrode for 12 h at room temperature in a humid chamber to promote strong Au-S bond formation. After incubation, the electrode was immersed in a 5 µM solution of 11-mercapto-1-undecanol (MCU) for 45–60 min at 37 °C to passivate any remaining gold surface, reducing non-specific adsorption and enhancing the biosensor’s performance. The electrodes were then rinsed with buffer solution and stored at −20 °C until further use.

### 2.3. Cuvette Design and Saliva Sample Handling

The biosensor system incorporated a custom-designed cuvette to house the saliva sample and electrode. The cuvette was designed with the electrode positioned facing downward to prevent interference from debris and other contaminants present in unfiltered saliva. This orientation allowed larger particles, such as epithelial cells and food remnants, to settle at the bottom of the cuvette, ensuring that the active sensing surface remained exposed to the liquid phase of the sample.

**Figure 1:**
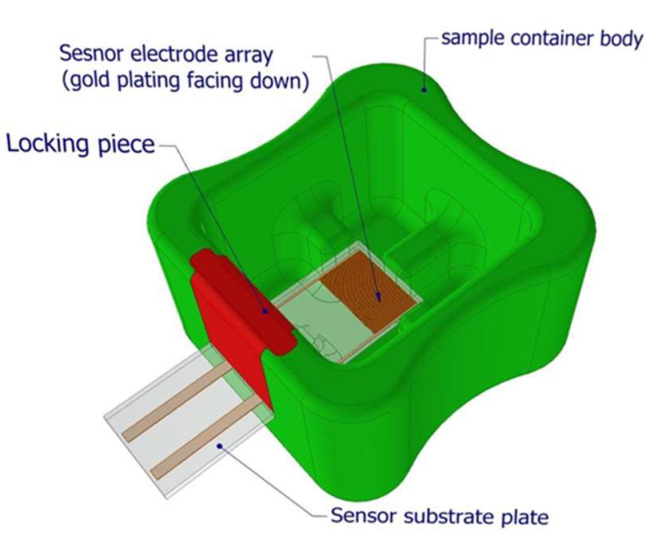
The cuvette and electrode setup in the biosensor. This figure displays the detailed design of the biosensor’s cuvette, including the downward-facing electrode configuration. The design allows saliva particles to settle at the bottom, preventing interference with the sensor’s active surface. The electrode is submerged in a saliva-buffer solution mix, ensuring that debris does not obstruct the impedance measurements. The ergonomic and cost-effective construction of the cuvette is also highlighted. Prototyping by 3D printing. Easily adaptable to mass production by injection molding.

**Figure 2:**
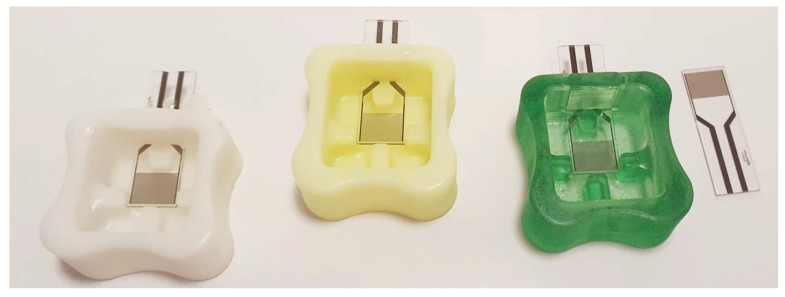
The cuvette and electrode setup in the biosensor and free IDE used and designed within the measurements.

The cuvette was fabricated using 3D printing technology (Prusa MK3s) from PolyEthylene Terephthalate Glycol (PET-G), chosen for its biocompatibility and ease of mass production (Figure 1 and Figure 2). The final design was modeled in Autodesk Inventor and optimized for ergonomic handling, allowing easy insertion of the electrode and consistent sample volume control. The dimensions of the cuvette were standardized to hold 1 mL of a saliva-buffer solution mixture, providing a stable environment for accurate and repeatable impedance measurements.

### 2.4. Buffer Preparation

The buffer solution used in this study was optimized to maintain stable ionic strength and promote efficient hybridization between the target RNA and the immobilized probe. The buffer composition was as follows: 1 µM Tris-HCl, 0.1 µM EDTA, and 100 µM MgSO_4_. All reagents were obtained from Merck (Darmstadt, Germany) and were of molecular biology grade. The buffer solution was prepared in large quantities to accommodate multiple experiments, ensuring consistency in measurements.

The ionic strength of saliva typically is around 40 mM, depending on individual factors such as diet, hydration, and health. This value is influenced by the presence of various ions, including sodium, potassium, calcium, chloride, bicarbonate, and phosphate, which are the primary contributors to the ionic environment of saliva [18]. The buffer used in our experiments is characterized by very low ionic strength (~100 µM) to ensure the stability of DNA and RNA (genetic material) and to maintain non-faradaic processes. The variability in salt concentration and composition of saliva can interfere with these measurements. However, saliva is typically diluted approximately 10-fold with the buffer, resulting in a final ionic strength of the solution in the range of ~4–8 mM. This dilution helps standardize the ionic environment and minimize measurement inconsistencies.

The low ionic strength of the buffer, when combined with saliva to reach approximately 4 mM, has a limited impact on Rsol during EIS measurements. This ionic strength is sufficient to ensure stable and reproducible results without significant fluctuations in Rsol between individuals, thus minimizing the variability in the impedance measurements. The buffer composition, therefore, does not substantially affect the solution resistance, allowing for reliable data analysis.

### 2.5. RNA Detection via Impedance Spectroscopy

Electrochemical impedance spectroscopy (EIS) was employed as the primary detection method for this study. EIS measures the impedance of the electrode surface as a function of frequency, providing detailed information on the interactions between the probe and target RNA. In this system, the binding of SARS-CoV-2 RNA to the immobilized probe altered the impedance, which was then used to quantify the concentration of viral RNA in the sample.

Measurements were conducted using a SensitSmart potentiostat (Palmsens, Houten, The Netherlands) set at a frequency of 20 Hz with a root mean square (RMS) voltage of 20 mV. The procedure for impedance measurement involved the following steps:

1. Blank Measurement: The electrode was immersed in 1 mL of buffer solution within the cuvette for 15 min. After this incubation, a blank impedance measurement was recorded to establish a baseline.
2. Sample Addition: The buffer was discarded, and a saliva sample consisting of 100 µL saliva mixed with 900 µL of buffer solution was added to the cuvette. The sample was incubated for 15 min to allow hybridization between the probe and any viral RNA present in the saliva.
3. Measurement: Following incubation, impedance was measured again. The change in impedance from the blank measurement was analyzed to determine the presence of viral RNA.
4. Cleaning: After each measurement, the electrode was cleaned using a 1 mM sodium hydroxide (NaOH) solution and rinsed with ultrapure water to prepare it for subsequent experiments.

**Figure 3:**
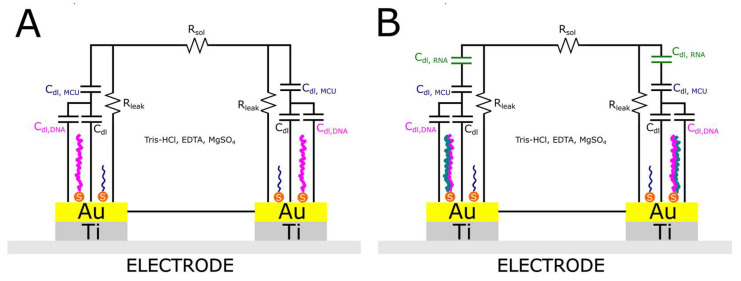
Panel (**A**,**B**) displays the equivalent circuits for stages blank and RNA measurement setups, respectively. The parameter *R*sol represents the intrinsic resistance of the buffer solution; R_leak_ is an equivalent resistance signifying a leakage current across the electrode–electrolyte interface; C_dl_ denotes the double layer capacitance formed between the IDEs and the adjacent buffer due to the applied voltage; C_dl,RNA_ indicates the change in double layer capacitance after probe immobilization. Note: MCU acts as an insulating layer that repels water and mobile ions away from the electrode into the solution the same as RNA/DNA duplex should further repel ions from the electrode surface, leading to additional reductions in double layer capacitance.

To measure the capacitance, a non-faradaic current method was employed, where a 0 V DC bias voltage was applied across the IDE sensor. An AC voltage of 20 mV root mean square (RMS) at a frequency of 20 Hz was also applied to the IDE sensors. Non-faradaic current, which flows without any charge transfer reactions, is primarily due to the charging and discharging of the electrical double layer at the electrode–electrolyte interface. The 0 V DC bias ensures no net direct current flows through the system, thus preventing faradaic processes like oxidation or reduction. The application of an AC voltage, instead of a constant DC voltage, allows for the study of the capacitive properties of the system. In this case, an AC voltage with an amplitude of 20 mV RMS was utilized. The variation in double-layer capacitance resulting from target binding is significantly influenced by the frequency of the applied AC signal. This capacitance, which forms at the electrode–electrolyte interface due to the displacement of mobile ions in the solution, decreases gradually with an increase in the frequency of the stimulus signal [19,20,21]. This process and schematic representation of the electrode before and after RNA interaction is shown in Figure 3.

In order to measure the capacitance, there is no need to measure the resistance (R) directly, as it is negligible compared to the reactance (X). The capacitance can be directly calculated from impedance measurements. To determine the capacitance change, impedance Z_o_ was measured before RNA binding, and impedance Z_1_ was measured after RNA binding. Impedance Z consists of a real part (resistance R) and an imaginary part (X), expressed as Z = R + jXZ = R + jXZ = R + jX. The imaginary part, X, which is related to the capacitance of the system, was extracted from these impedance measurements. The capacitance was calculated from the reactance using the formula C = 1/2πf∣X∣, where f is the frequency of the applied AC signal (20 Hz). Therefore, the change in capacitance, which directly results from impedance measurements, does not require the separate measurement of resistance [19,20,21].

**Figure 4:**
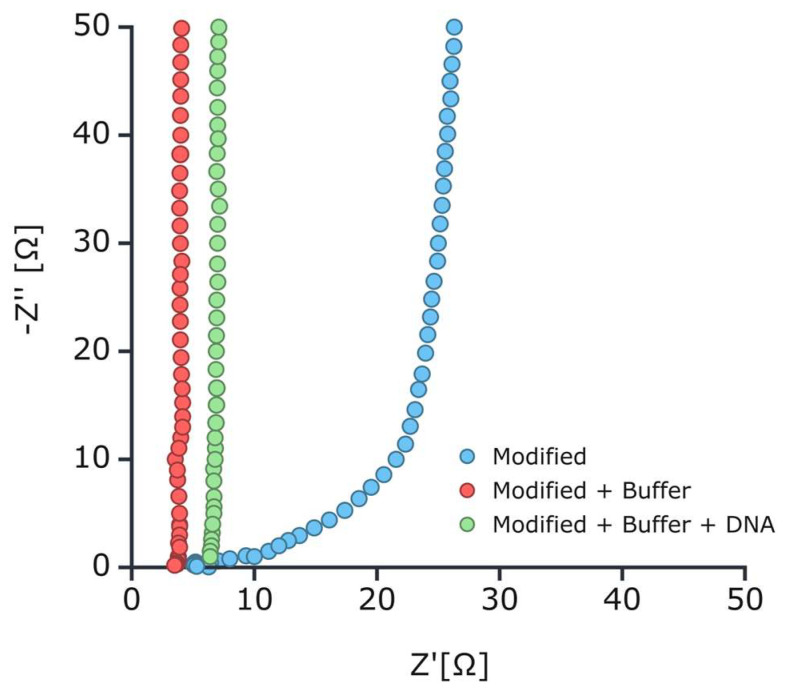
The Nyquist plot presented shows the impedance characteristics of the IDE modified with DNA molecules under different conditions: (i)—Modified without buffer (blue): This curve represents the impedance of the dry modified electrode. (ii)—Modified + Buffer (red): Adding buffer to the modified electrode decreases the impedance, as shown by the shift of the curve to the left. (iii)—Modified + Buffer + DNA (green): When DNA complementary to the modified surface is added to the buffer at 10 µM concentration.

Electrochemical Impedance Spectroscopy measurements were performed, and the results were plotted as a Nyquist plot. The measurements were conducted with use of SensiSmart (Dropsens) over a frequency range from 0.01 Hz to 200 kHz. For clarity, only a fragment of the Nyquist plot, specifically in the range of Z_Re_ and Z_im_ 50/50 Ohm, is presented in Figure 4. The results clearly show that Z_im_ is much higher than Z_re_ for different frequences. It suggests a unique impedance profile that can be attributed to the system’s characteristics, such as the high capacitive contribution from the double layer and the relatively high solution resistance (R_sol_) due to the low ionic strength of the buffer. The Nyquist plot (Figure 4) for the dry IDE modified with DNA, measured dry, and in a solution containing 1 µM Tris-HCl, 0.1 µM EDTA, and 100 µM MgSO_4_, exhibits unusual behavior, not fitting the typical semicircular shape observed in EIS for systems involving charge transfer reactions. The impedance values, with Z_re_ ranging from <100 Ω for dry measurement and <30 Ω for buffered systems and Z_im_ reaching up to 150,000 Ω, indicate that the impedance is primarily governed by capacitive and resistive effects, rather than electrochemical processes (see buffer composition). Key factors influencing the impedance behavior could be as follows: (i) The absence of electrochemical processes—the absence of significant electrochemical reactions (such as charge transfer) leads to the lack of the typical semicircular response that would otherwise arise from Faradaic processes, as stated before and in the literature [19,20,21], we are expecting non-faradic process. The lack of such processes results in a predominantly capacitive response, which explains the high imaginary impedance (Z_im_) at low frequencies. (ii) Solution resistance (R_sol_)—the low ionic strength of the buffer (1 µM Tris-HCl, 0.1 µM EDTA, 100 µM MgSO_4_) causes a relatively high solution resistance (R_sol_). This is evident in the real component of the impedance (Z_re_), which ranges between 0 and 100 Ω, as the low ion concentration restricts the current flow through the solution. The high value of Zim (up to 150,000 Ω) is likely a result of the significant capacitive effects that dominate at lower frequencies, as the impedance from the double layer capacitance (C_dl_) is prominent in this low-ionic-strength environment. Finally, (iii) double layer capacitance (C_dl_)—the Tris-HCl buffer, in combination with magnesium ions and DNA, creates a stable double layer at the electrode surface, which contributes significantly to the overall impedance. This capacitive behavior is dominant in this system, leading to a large imaginary impedance (Zim). The shape of the Nyquist plot suggests a system where the impedance is largely capacitive at lower frequencies, without the formation of a well-defined semicircle. Moreover, EDTA, as a chelating agent, prevents complexation of the magnesium ions, ensuring their stable interaction with the electrode surface and DNA. This further stabilizes the electric double layer but does not contribute to any Faradaic current, thus, maintaining the capacitive nature of the impedance response. All in all, the observed Nyquist plot indicates a system dominated by solution resistance (R_sol_) and the double layer capacitance (C_dl_), without significant electrochemical or charge transfer processes. The high imaginary component of the impedance at low frequencies reflects the capacitive nature of the system, while the real part remains relatively small due to the limited ionic conductivity in the low-ionic-strength buffer. This leads to an atypical impedance response that does not exhibit the characteristic semicircle of systems with electrochemical reactions but instead shows a more extended or exponential-like shape in the Nyquist plot.

### 2.6. Sensitivity and Specificity Evaluation

To evaluate the biosensor’s performance, sensitivity and specificity tests were conducted using synthetic SARS-CoV-2 RNA (ATCC-VR-3276SD, LGC Standards, Warsaw, Poland) at concentrations ranging from 10^1^ to 10^6^ copies per µL. Negative controls without RNA were also included to assess the biosensor’s ability to avoid false positive results. The sensitivity of the biosensor was defined as the lowest detectable concentration of RNA, while the specificity was determined by its ability to differentiate between SARS-CoV-2 RNA and non-target sequences. Each measurement was repeated using 10 different electrodes to ensure reproducibility. A linear standard curve was established by plotting the impedance changes against the logarithm of RNA concentration, and the threshold for positive detection was set based on a capacitance of less than 650 nF. Capacitance values between 650 and 750 nF were considered uncertain, while those above 750 nF indicated a negative result. The Limit of Detection (LOD) for the analytical method was calculated using a stepwise approach starting with the background signal measurement. For this, the capacity values (in nF) were recorded at 0 and up to 10^6^ copies/µL RNA concentration. Next, a calibration curve was constructed to relate RNA concentration (in terms of the number of copies) to the corresponding mean capacity values. The concentrations tested ranged from 0 to 1,000,000 RNA copies. For each concentration, the mean capacity was calculated, and a logarithmic relationship between RNA concentration and capacity was assumed. To model this, the data were fitted using linear regression, where the RNA concentrations were expressed as the logarithm of the number of copies (log_10_), and the capacity values were the dependent variable. The resulting regression equation was: Capacity = a+b⋅log_10_ (Concentration). To calculate the LOD in terms of RNA concentration, we used the formula for the detection limit based on the slope of the calibration curve: LOD (Concentration) = SD_a_/(a × 3.3), where SD_a_ is the standard deviation of the signal and a is the slope of the calibration curve. The slope of the calibration curve, obtained from the linear regression analysis, was SD_a_ = 5.94792517 a = −77.053571, giving LOD = 0.55 copies of RNA/µL (~1 aM).

### 2.7. Saliva Collection and RT-qPCR Cross-Validation

Saliva samples were collected from 60 volunteers aged 20–67 years, with both positive and negative RT-qPCR-confirmed SARS-CoV-2 status. Throat swabs were collected and analyzed using the respiraSC2 multi-RT-PCR Kit (Qiagen, Gdansk, Poland) following the manufacturer’s protocol. After saliva collection, the volunteers were instructed to use the biosensor testing kit, which included the potentiostat, electrodes, and buffer solution. The results of the biosensor measurements were compared to the RT-qPCR outcomes to assess the accuracy, sensitivity, and specificity of the biosensor. Ethical approval for this study was obtained from the bioethical committee (NKBBN/162/2022).

### 2.8. Statistical Methods

Data analysis was conducted using regression techniques to evaluate the correlation between impedance changes and RNA concentrations. The sensitivity and specificity of the biosensor were calculated based on true positive, false positive, true negative, and false negative results obtained from cross-validation with RT-qPCR. All experiments were performed in triplicate, and statistical significance was determined using *p*-values < 0.05.

## 3. Results

### 3.1. Laboratory Validation of the Biosensor

The biosensor’s performance was first evaluated in a controlled laboratory setting using synthetic SARS-CoV-2 RNA at concentrations ranging from 10^1^ to 10⁶ copies/µL. Impedance changes were measured as a function of RNA concentration to assess the sensitivity and accuracy of the biosensor. The resulting data demonstrated a clear relationship between RNA concentration and changes in impedance, with higher RNA concentrations producing significant decreases in the measured capacitance. The distribution of capacitance values for different RNA concentrations demonstrated a clear trend, with lower capacitance values correlating with higher RNA concentrations.

**Figure 5:**
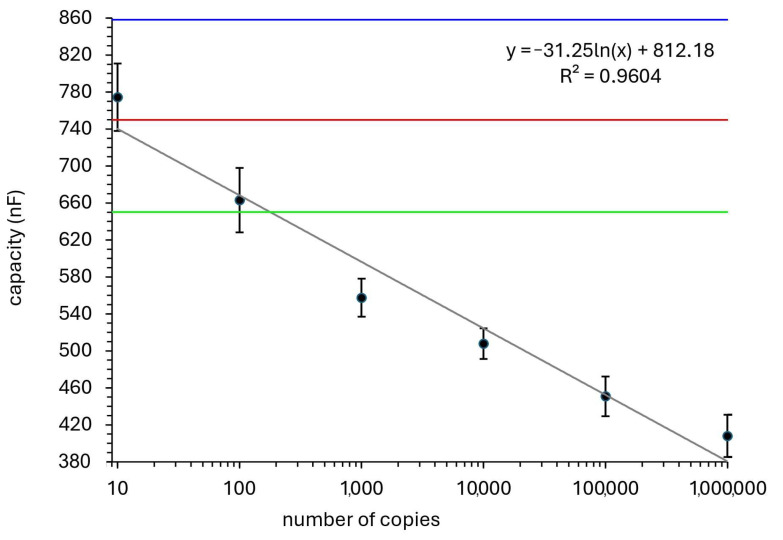
Regression analysis of capacitance changes versus RNA concentrations. The figure shows a regression plot illustrating the linear relationship between the changes in capacitance and different oligonucleotide concentrations. The data confirms the linearity of the biosensor’s response, with a strong correlation (R^2^ = 0.96) between impedance changes and the logarithmic concentration of RNA. The lines represent: the capacity for blank sample (0 RNA copies; 859.8 nF)—blue line; positive result threshold (650 nF)—green line; negative result threshold (750 nF)—red line.

The blank measurements, taken using only the buffer solution, consistently resulted in capacitance values around 800 nF, indicating no binding of RNA to the sensor surface. In contrast, the highest concentration of RNA (10⁶ copies/µL) produced capacitance values as low as 400 nF, demonstrating the biosensor’s capability to detect substantial changes in RNA levels. The standard curve was constructed by plotting the capacitance values against the logarithmic RNA concentrations, yielding a linear correlation with an R^2^ value of 0.96, indicating excellent accuracy in quantifying viral RNA (Figure 5).

Capacitance Threshold for Detection: Based on the collected data, we established the following thresholds for interpreting the biosensor’s results:

- Positive Result: Capacitance values below 650 nF were consistently associated with the presence of SARS-CoV-2 RNA.
- Negative Result: Capacitance values above 750 nF indicated the absence of viral RNA.
- Uncertain Results: Values between 650 and 750 nF were classified as uncertain and required additional testing.

These thresholds were cross-validated with RT-qPCR, demonstrating that the biosensor accurately classified positive and negative samples with minimal ambiguity.

#### 3.1.1. Sensitivity and Specificity of the Biosensor

**Figure 6:**
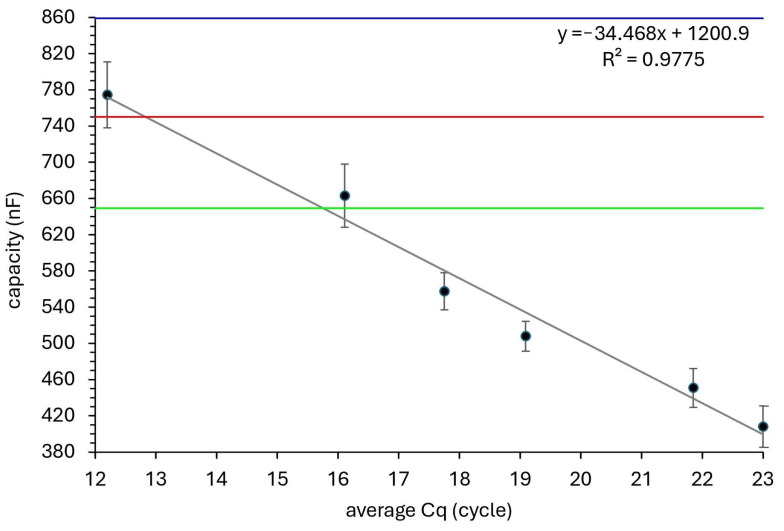
Comparison of RT-qPCR results with biosensor capacitance changes across different SARS-CoV-2 RNA concentrations. This figure presents a scatter plot comparing the RT-qPCR Cq values with the capacitance changes measured by the biosensor. The fitting curve shows excellent agreement between the two methods, further validating the biosensor’s performance for detecting viral RNA. This correlation emphasizes the biosensor’s accuracy in measuring viral load. The lines represent the capacity for blank sample (0 RNA copies; 859.8 nF)—blue line; positive result threshold (650 nF)—green line; negative result threshold (750 nF)—red line.

The biosensor’s sensitivity was determined by evaluating its ability to detect low concentrations of SARS-CoV-2 RNA. Remarkably, the biosensor was able to reliably detect RNA at concentrations as low as 1 aM (approximately 10^1^ copies/µL). Even at these low concentrations, the impedance changes were sufficient to differentiate between the blank and RNA-containing samples (Table S2 and Figure 6).

#### 3.1.2. Reproducibility and Specificity

Each RNA concentration was measured in triplicate using 10 different biosensors to assess reproducibility. The capacitance values across different biosensors remained consistent, with minimal variation between measurements. The coefficient of variation (CV) for the biosensor measurements was less than 5%, indicating high precision.

Specificity was evaluated by testing the biosensor’s response to non-target RNA sequences. Negative control samples, which lacked SARS-CoV-2 RNA, consistently produced capacitance values above 750 nF, confirming that the biosensor did not generate false positives. Additionally, no cross-reactivity was observed with other viral RNA sequences, further validating the biosensor’s high specificity.

#### 3.1.3. Saliva Sample Testing

**Figure 7:**
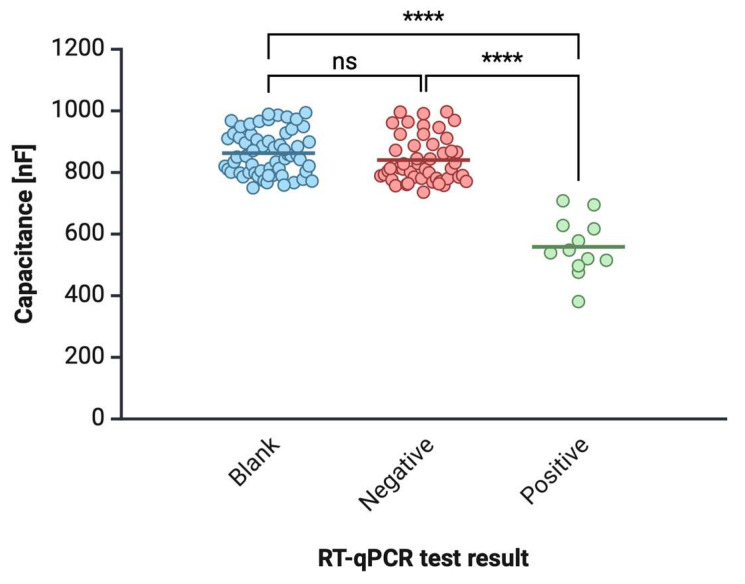
Comparison of RT-qPCR results (positive, negative, blank) with biosensor capacitance changes across different patient samples, with the ANOVA statistics. The blank sample and negative result (absence of viral RNA) were non-statistically significant, while the Positive vs. Negative and Positive vs. Blank revealed *p* < 0.001; “ns”—not statistically significant, “****”—*p* < 0.001.

Saliva samples from 60 volunteers were tested using the biosensor, and the results were compared with RT-qPCR, the gold standard in COVID-19 diagnostics. Among the 60 participants, 12 individuals were tested positive for SARS-CoV-2 via RT-qPCR, and 48 individuals were tested negative (Figure 7). Table S1 in Supplementary Materials displays a sample of results obtained from 60 volunteers, showing the blank capacitance values, the measured capacitance values after saliva testing, and the corresponding RT-qPCR result. The biosensor successfully detected all 12 positive cases, with capacitance values ranging from 450 to 600 nF, well within the established positive threshold. All 48 negative cases were correctly identified, with capacitance values exceeding 750 nF. There were no false positives or false negatives, resulting in 100% sensitivity and specificity in this cohort. These results were consistent with the laboratory validation, confirming the biosensor’s accuracy in real-world conditions.

### 3.2. Cross-Validation with RT-qPCR

To ensure the reliability of the biosensor’s performance, all results were cross-validated with RT-qPCR (Table 1 and Table 2). The amplification curves from RT-qPCR showed strong correlation with the capacitance measurements recorded by the biosensor.

As shown in Figure S1 in Supplementary Materials, the RT-qPCR curves demonstrated typical amplification patterns for the SARS-CoV-2 genes (ORF1ab, N, and E), with cycle threshold (Cq) values corresponding to viral load, which aligned with biosensor measurements.

For example, samples with Cq values of 18 to 23 (indicating moderate viral loads) produced capacitance values between 500 and 600 nF on the biosensor. Samples with higher Cq values (indicating low viral loads) produced capacitance values closer to 650 nF, reflecting the lower concentrations of viral RNA. This alignment between the two methods provides further evidence of the biosensor’s ability to detect and quantify viral RNA with a high degree of accuracy.

### 3.3. Point-of-Care Testing Results

The biosensor’s usability and practicality were evaluated through point-of-care (POC) testing conducted by volunteers. Each participant received a test kit consisting of a biosensor, a potentiostat with a smartphone, and a buffer solution. The volunteers performed 7 to 10 tests with each biosensor, collecting saliva samples and measuring capacitance values (the same biosensor was used until the first positive result). Results of these tests were compared to laboratory RT-qPCR analysis.

In all cases, the biosensor results aligned with RT-qPCR findings. Volunteers reported ease of use, and the majority of participants rated the biosensor highly for its simplicity, ergonomics, and size. Participants also noted that the biosensor’s rapid response time (typically within 15 min) made it an ideal tool for POC applications.

### 3.4. Statistical Analysis

The statistical analysis confirmed the robustness of the biosensor across all tested conditions. The standard curve derived from the impedance measurements showed a linear response across a wide range of RNA concentrations (10^1^ to 10⁶ copies/µL), with high reproducibility (R^2^ = 0.98). The overall sensitivity of the biosensor was calculated to be 100%, with specificity also at 100%, based on the cross-validation with RT-qPCR results.

The statistical significance of the results was further validated by the *p*-values, which were all below 0.05, confirming that the differences in capacitance values between positive and negative samples were statistically significant. This strong statistical correlation between the biosensor’s capacitance changes and viral RNA concentration highlights the reliability of this technology for real-time viral detection.

## 4. Discussion

This study presents a novel, reusable biosensor for the direct detection of SARS-CoV-2 RNA from unfiltered saliva using electrochemical impedance spectroscopy (EIS). The biosensor, designed to operate without the need for sample filtration or complex preparation, demonstrated excellent sensitivity and specificity, making it a promising tool for point-of-care (POC) diagnostics. The performance of the biosensor, validated both in laboratory conditions and real-world settings, highlights its potential as a rapid, cost-effective alternative to RT-qPCR for COVID-19 detection. The developed bioanalytical microsystem addresses several critical limitations of existing SARS-CoV-2 RNA detection technologies by incorporating an innovative cuvette design and inverted electrode configuration.

These advancements, in combination with robust electrochemical and RT-qPCR methodologies, significantly enhance the sensitivity and specificity of the system, particularly in challenging sample matrices such as saliva. Overcoming limitations of conventional traditional electrochemical sensors often suffers from signal interference caused by environmental contaminants, including particulate matter and organic debris, especially when applied to complex biological samples like saliva [22,23,24].

Saliva, a preferred medium for non-invasive diagnostics, contains epithelial cells, microbial communities, and a diverse range of organic molecules, which can impair sensor performance [25]. Studies have highlighted the difficulty of obtaining reproducible and accurate electrochemical signals in such matrices due to biofouling and non-specific adsorption on electrode surfaces [19,20,21]. Our microsystem effectively mitigates these challenges through its innovative cuvette design, which creates a controlled microenvironment for electrochemical measurements. The inverted electrode configuration further reduces the impact of airborne contaminants by preventing direct exposure of the sensing surface to the ambient environment. This strategic modification ensures stable electrode performance, even in samples with high organic content, as demonstrated in saliva-based SARS-CoV-2 RNA detection experiments. The system maintained high reproducibility and linearity of signal response, as indicated by the robust logarithmic correlation between RNA concentration and capacitance (Figure 3). Saliva-based diagnostics are increasingly recognized for their practicality in mass testing scenarios, yet they are notoriously challenging due to the sample’s heterogeneity. Previous studies have reported significant signal distortions arising from cellular debris and enzymatic activities inherent in saliva [26]. Our system effectively circumvented these issues by leveraging the enclosed cuvette structure, which minimizes contamination risks during sample handling and measurement. Additionally, the electrode surface’s enhanced biocompatibility, achieved through optimized immobilization protocols, allowed for accurate RNA detection despite the presence of interfering substances.

The microsystem’s sensitivity, achieving detection limits as low as 1 aM, surpasses many reported systems employing conventional electrodes [21]. By integrating electrochemical detection with RT-qPCR validation, our platform ensures consistency with established diagnostic standards while offering the scalability and speed required for point-of-care applications. These findings align with prior reports on capacitance-based biosensing, where effective probe immobilization and stable surface properties are critical for achieving high sensitivity [18].

The design advancements presented in this study underscore the system’s potential for real-world deployment in non-invasive diagnostic settings. By eliminating contamination-related signal variability and ensuring compatibility with complex biological matrices, the microsystem not only enhances diagnostic accuracy but also broadens the applicability of electrochemical sensors in public health initiatives. Future developments could focus on expanding the system’s capabilities to detect multiple analytes simultaneously, further increasing its utility in multiplexed diagnostic workflows. In conclusion, this study demonstrates that the integration of a novel cuvette and inverted electrode design can overcome key limitations associated with traditional biosensors, particularly in applications involving complex biological samples like saliva. By addressing these challenges, the proposed microsystem provides a robust platform for early and accurate detection of SARS-CoV-2 RNA, setting a benchmark for future advancements in bioelectrochemical diagnostics.

The impedance behavior of the sensor observed in this study deviates from the typical patterns commonly seen in electrochemical systems where charge transfer processes dominate. However, this non-standard impedance response aligns with the design goals of the sensor. The sensor was intentionally developed to operate in a non-Faradaic regime, where electrochemical reactions are minimized (which could lead to RNA damage), and the system’s response is governed predominantly by capacitive effects. This design choice was made to emphasize the sensitivity to surface interactions, particularly in systems where electrochemical reactions might interfere with or obscure the primary signal from the sensor’s surface, such as in biosensing applications. The buffer composition and the very low ionic strength were carefully chosen to limit ionic conductivity, effectively isolating the sensor from electrochemical noise and minimizing Faradaic processes. By ensuring that the sensor operates in a predominantly capacitive mode, the aim was to focus on the interactions at the electrode surface—such as the formation of a stable electric double layer and the behavior of DNA-modified electrodes in a non-electrochemical environment. The resulting impedance profile, dominated by double layer capacitance (C_dl_), reflects this design approach. This non-standard impedance response is not a limitation but rather a strength of the sensor, as it ensures the system is highly sensitive to surface phenomena. The Nyquist plot observed, with a high imaginary component (Z_im_) and a relatively low real component (Z_re_), highlights the capacitive nature of the system and its resistance to electrochemical interference. This design allows for a more accurate and specific response to biomolecular interactions, making it particularly suitable for applications such as DNA detection, where non-Faradaic interactions are often the primary source of signal. Thus, while the impedance behavior may appear atypical compared to systems with prominent Faradaic processes, it aligns perfectly with the sensor’s intended application and design principles, reinforcing its potential for sensitive and specific detection in non-electrochemical environments.

Cross-validation with RT-qPCR, the widely accepted standard for SARS-CoV-2 detection, revealed strong agreement between the two methods. The biosensor consistently produced capacitance values that correlated well with the RT-qPCR Cq values, indicating its ability to quantify viral RNA with high precision. Samples with higher viral loads, indicated by lower Cq values in RT-qPCR, produced lower capacitance values on the biosensor, confirming the direct relationship between RNA concentration and impedance changes.

The use of electrochemical impedance spectroscopy in biosensing has proven to be highly effective for detecting biomolecular interactions, including viral RNA hybridization [27,28]. In this study, EIS allowed us to measure the impedance changes that occur when SARS-CoV-2 RNA binds to the oligonucleotide probe immobilized on the electrode surface. This label-free detection method offers several advantages over traditional techniques, such as fluorescence-based or enzyme-linked assays or other new methods [29,30,31], which require secondary reagents and additional steps.

One of the key advantages of the proposed biosensor is its reusability, aligning with the principles of environmental sustainability [32,33,34]. Unlike single-use diagnostic devices, which generate significant amounts of biomedical waste, this biosensor offers a long-term, eco-friendly alternative [35,36,37]. The hydrophobic coating on the electrode surface not only enhances performance by preventing air bubble formation and salt crystal deposition but also extends the device’s operational lifespan across multiple testing cycles. This durability reduces the volume of disposable materials typically associated with diagnostics, contributing to a lower environmental footprint. The simple and efficient cleaning protocol—using NaOH solution and ultrapure water—further underscores its environmental benefits. This method minimizes the use of hazardous chemicals and streamlines the reconditioning process, ensuring the biosensor is ready for reuse without compromising accuracy or sensitivity. In high-throughput environments, such as hospitals or testing facilities, the ability to reuse a biosensor reduces waste generation, operational costs, and the demand for raw materials. These factors collectively address critical environmental concerns associated with the diagnostic industry, which is often criticized for its contribution to chemical waste and single-use plastics. This innovation demonstrates how modern medical devices can prioritize both performance and ecological responsibility, offering a model for future developments in diagnostic technology. Through its reusability and minimal waste generation, the biosensor represents a step forward in harmonizing healthcare advancements with the pressing need for environmental sustainability.

The results of the point-of-care (POC) testing conducted by volunteers further validated the practicality of the biosensor in real-world scenarios. Volunteers were able to easily use the biosensor kits, and the results aligned perfectly with RT-qPCR outcomes. This user-friendly aspect is crucial for POC diagnostics, particularly in decentralized or low-resource environments where specialized laboratory equipment may not be available. The biosensor’s rapid response time, minimal sample handling, and ergonomic design make it an ideal candidate for widespread deployment in healthcare settings, community testing, and even home use. Feedback from volunteers also highlighted the importance of the biosensor’s intuitive design. Most participants reported that they found it easy to operate. This is a critical factor for POC devices, where non-expert users must be able to perform tests accurately and obtain reliable results without extensive training. The combination of user-friendliness, rapid detection, and high accuracy positions this biosensor as a versatile tool for POC applications in various healthcare contexts.

### Limitations and Future Directions

While the biosensor demonstrated exceptional performance in this study, there are some limitations that should be addressed in future research. First, although the sample size of 60 volunteers provided robust data, larger-scale studies with more diverse populations are necessary to further validate the biosensor’s performance. Additionally, while the biosensor exhibited high sensitivity and specificity for detecting SARS-CoV-2 RNA, its application to other pathogens should be explored. By modifying the oligonucleotide probes, the biosensor could potentially be adapted to detect a wide range of viral, bacterial, and fungal infections. Another area for future improvement is the miniaturization of the biosensor and the integration of wireless communication technologies to enable remote data transmission. This could facilitate real-time monitoring of infectious diseases, enabling faster response times in outbreak scenarios. Furthermore, integrating the biosensor with smartphone platforms could provide a powerful tool for at-home diagnostics, making viral testing more accessible to the general population.

## 5. Conclusions

In conclusion, this study introduces a highly sensitive, reusable biosensor that offers a rapid, accurate, and cost-effective alternative to traditional diagnostic methods such as RT-qPCR. By leveraging electrochemical impedance spectroscopy and an innovative cuvette design, the biosensor effectively detects SARS-CoV-2 RNA from unfiltered saliva samples without the need for extensive sample preparation. Its high sensitivity, specificity, reusability, and ease of use make it a promising tool for point-of-care diagnostics, particularly in settings where rapid and reliable viral detection is critical. With further validation and development, this biosensor has the potential to revolutionize diagnostic testing, not only for COVID-19 but also for a broad spectrum of infectious diseases. Its adaptability, combined with its user-friendly design, positions it as a valuable asset in the ongoing efforts to improve global public health.

The high sensitivity of EIS arises from its ability to detect small changes in the electrical properties of the electrode–sample interface. In the case of viral RNA detection, the impedance changes are proportional to the amount of RNA bound to the probe, providing a direct quantitative measurement of viral load. Additionally, the simplicity of the EIS setup makes it ideal for integration into portable, user-friendly devices suitable for POC testing. By utilizing EIS, we were able to eliminate the need for complex signal amplification steps, further streamlining the diagnostic process.

Although gold standard RT-qPCR is highly sensitive, it has several limitations, including the need for specialized laboratory infrastructure, time-intensive sample preparation, and a multi-step amplification process. The biosensor, by contrast, offers a simplified and rapid diagnostic solution that can deliver results in less than 15 min, without the need for RNA extraction or amplification. This time efficiency makes the biosensor particularly suitable for use in resource-limited settings or during high-demand scenarios, such as large-scale screening during pandemics.

## 6. Patents

Patent was granted P.436020 (2023) [38].

## Figures and Tables

**Table 1:** Amplification conditions for RT-qPCR analysis of SARS-CoV-2 RNA in saliva samples. This table outlines the specific steps and conditions used in RT-qPCR to detect SARS-CoV-2 RNA in saliva. The table includes temperatures, time per cycle, and the number of cycles for each step, including reverse transcription, denaturation, amplification, and cooling.

Step	Temperature [°C]	Time [s]	Number of Cycles
Reverse transcription	55	180	1
Denaturation	95	15	1
Amplification	95	15	50
	58	30	
Cooling	40	30	1

**Table 2:** Concentration of SARS-CoV-2 RNA and corresponding cycle threshold (Cq) values from RT-qPCR analysis. This table lists the RNA concentrations tested (in ng/μL), the corresponding number of viral RNA copies, and the average Cq values obtained from RT-qPCR. The data demonstrate how the viral load correlates with the RT-qPCR amplification cycle numbers.

Concentration RNA [ng/µL]	Copies [Number]	Average Cq [Cycles]
10^−1^	10^6^	12.45
10^−2^	10^5^	16.36
10^−3^	10^4^	18.00
10^−4^	10^3^	19.34
10^−5^	10^2^	22.10
10^−6^	10^1^	23.25

## Data Availability

The data will be available upon request.

## Supplementary Materials

The following supporting information can be downloaded at: https://www.mdpi.com/article/10.3390/s25020360/s1, The Supplementary Materials contain the comparative analysis, which allowed for us to select the sequence of probe. Table S1 displays a sample of results obtained from 60 volunteers, showing the blank capacitance values, the measured capacitance values after saliva testing, and the corresponding RT-qPCR result. Figure S1: RT-qPCR representative amplification curves for SARS-CoV-2 genes. Table S2 displays the standard curve results obtained from laboratory measurements with use of different electrodes.
