# Supplementary material for: Reusable Biosensor for Easy RNA Detection from Unfiltered Saliva
Source: Sensors (Basel). 2025 Jan 9;25(2):360. doi: 10.3390/s25020360 (PMC11769206; doi:10.3390/s25020360)
Supplement: Supplementary file 1 [file sensors-25-00360-s001.zip › sensors-3300971-supplementary.pdf]

Supplementary Material

Reusable Biosensor for Easy RNA Detection from Unfiltered Saliva

Paweł Wityk [1,2,3,*], Agata Terebieniec [4], Robert Nowak [5,6], Jacek Łubiński [7], Martyna Mroczyńska-Szeląg [8], Tomasz Wityk [1] and Dorota Kostrzewa-Nowak [9]

1 Map Your DNA Ltd., Świerkowa 40, 83-330 Lniska, Poland; info@mapyourdna.eu
2 Beckman Institute for Advanced Science and Technology, University of Illinois at Urbana-Champaign, Urbana, IL 61801, USA; pwityk@illinois edu, ORCID: 0000-0001-8612-727X
3 Department of Biopharmaceutics and Pharmacodynamics, Medical University of Gdańsk, Al. Gen. J. Halera 107, 80-416 Gdańsk, Poland
4 Fungal Physiology, Westerdijk Fungal Biodiversity Institute and Fungal Molecular Physiology, Utrecht University, 3584 Utrecht, The Netherlands; terebieniecagata@gmail.com
5 Institute of Physical Culture Sciences, University of Szczecin, 40B Piastów Al., Building 6, 71-065 Szczecin, Poland; robert.nowak@pum.edu.pl
6 Department of Pathology, Pomeranian Medical University in Szczecin, 1 Unii Lubelskiej St., 71-242 Szczecin, Poland
7 Faculty of Mechanical Engineering and Ship Technology, Gdańsk University of Technology, Narutowicza 11/12, 80-233 Gdańsk, Poland; jacek.lubinski@pg.edu.pl
8 Faculty of Chemistry, Gdańsk University of Technology, Narutowicza 11/12, 80-233 Gdańsk, Poland; martyna.mroczynska@pg.edu.pl, ORCID: 0000-0002-6476-7580
9 Department of Clinical and Molecular Biochemistry, Pomeranian Medical University in Szczecin, 72 Powstańców Wlkp. Al., 70-111 Szczecin, Poland; dorota.kostrzewa.nowak@pum.edu.pl
* Correspondence: pwityk@illinois.edu

Abstract: Biosensors are transforming point-of-care diagnostics by simplifying the detection process and enabling rapid, accurate testing. This study introduces a novel, reusable biosensor designed for direct viral RNA detection from unfiltered saliva, targeting SARS-CoV-2. Unlike conventional methods requiring filtration, our biosensor leverages a unique electrode design that prevents interference from saliva debris, allowing precise measurements. The biosensor is based on electrochemical principles, employing oligonucleotide probes immobilized on a hydrophobic-coated electrode, which prevents air bubbles and salt crystal formation. During validation, the biosensor demonstrated a sensitivity and specificity of 100%, accurately identifying SARS-CoV-2 in saliva samples without false positives or negatives. Cross-validation with RT-qPCR, the gold standard for COVID-19 diagnostics, confirmed the reliability of our device. The biosensor's performance was tested on 60 participants, yielding 12 true positive results and 48 true negatives, aligning perfectly with RT-qPCR outcomes. This reusable, easy-to-use biosensor offers significant potential for point-of-care applications in various healthcare settings, providing a fast, efficient, and cost-effective method for detecting viral infections such as COVID-19. Its robust design, minimal sample preparation requirements, and multiple-use capability mark a significant advancement in biosensing technology.

```
                                                                ". . .."
MN908947.3      ----aacttgaacagccctatgtgttcatcaaacgttcggatgctcgaactgcacctcat
NC_006577.2     -----gttcatacaagactaaacgtt-----------------------atgttgtacat
NC_006213.1     tagaagtgtgtgtaacactggtcgttg--------cactgttaataagcatgtggcctat
NC_002645.1     ----------------------------------------------------------at
NC_005831.2     ----------------------------------------------------------tt
KJ361502.1      -aggtcctcgtgtatacctggttgagaggctca----------------ttgcttgtgaa

MN908947.3      agtcatttgacttaggcgacgagcttggcactgatccttatg-----------aagattt
NC_006577.2     atgattttagtgtagaagatgcttatgctgaggttcatgctgagcctaaaggtaaatatt
NC_006213.1     atgattttaaagttgaagatgcttatgaccaggtgcatgatgagcctaagggtaagtttt
NC_002645.1     aggaatttgatgt-----tgtcttcggtaaga----------------------------
NC_005831.2     aggactttgatgt-----tgtttttggcca------------------------------
KJ361502.1      acgattttgaggcggat---------------------------cctaaaggcaaatatg
                *    ***..  .

MN908947.3      tcaagaaaactggaacactaaacatagca----------gtggtgttacccgtgaactca
NC_006577.2     cacaaaaagcttatgctttacttagacaat------atcgtggtattaaacccgtact--
NC_006213.1     ctaagaaggcttatgctttaattagagggt------atcgtggtgttaaaccacttct--
NC_002645.1     ---------------------------------------gaggtggtggtaatgtgac--
NC_005831.2     ----------------------------------------tggtgcaggaagtgtggt--
KJ361502.1      cccagaa---------tctgcttaagaagttgattggcggtgatgtcac-----------
                                                         *.*.  .

MN908947.3      tgcgtgagcttaacggaggggcatacactcgctatgtcgataacaacttctgtggccctg
NC_006577.2     -------------------------------ttttgtagaccagtatggttgtgactatt
NC_006213.1     -------------------------------ctatgtagaccagtatggttgtgattata
NC_002645.1     -------------------------------atacactgaccagtatctctgtggcgccg
NC_005831.2     -------------------------------ttttgtggataagtacatgtgtggttttg
KJ361502.1      -------------------------------tccagttgaccaatacatgtgtggcgttg
                                                .  .. **. *  *.   ****..  .

MN908947.3      ---------------gaccttctagcacgtgctggtaaagcttcatgcactttgtccgaa
NC_006577.2     -----------------------agcttatggtcat------------------------
NC_006213.1     -----------------------ggcttatgctgat------------------------
NC_002645.1     -----------------------gatttatggcagtttgt--------------------
NC_005831.2     -----------------------aacatgtgggaatttag--------------------
KJ361502.1      gctgatgttgaagcggacgtcgcagcacgtgctgat------------------------
                                       ... ..**   .*

MN908947.3      caactggactttattgacac---taagaggggtgtatactg----------ctgccgtga
NC_006577.2     ----tattctttgcaagata---tgagacaaaagcagtctgtatggcttgccaattgtga
NC_006213.1     ----aagacattgcaagaaa---tgaaggcattatttcctacttggagtcaggaactcct
NC_002645.1     --tgaccatttcggtgagaa---cgaagaaattatcatcaa----------------tgg
NC_005831.2     --ggattactttaataataatactgatagtattgttattgg----------------tgg
KJ361502.1      gacgaaggcttcatcacatt---aaagaacaatctatatag-------------------
                        . *..  .        .* .  .   .   . .

MN908947.3      acatgagcatgaaattgcttggtacacggaacgttctgaaaagagctatgaattgcagac
NC_006577.2     ctttgatattgtagtggcttggcatgtagttcgtgat---------------------tc
NC_006213.1     ttttgatgtaattgtggcatggcatgttgtgcgtgat---------------------cc
NC_002645.1     tcatacttacgtttgtgcttggcttactaagcgtaa------------------------
NC_005831.2     tgtcacttatcaactagcatgggatgttatacgtaa------------------------
KJ361502.1      ------------attggtttggcatgttgagcgtaaa-----------------------
                                *. ***  ... .  ***
```

```
MN908947.3       tgttttctacaaagaaaacagttacacaacaaccataaaaccagttacttataaattgga
NC_006577.2      ctgcttgtat--------------------------------------------------
NC_006213.1      ttgtctgtat--------------------------------------------------
NC_002645.1      ctgtttgtattg------------------------------------------------
NC_005831.2      caacttgtataa------------------------------------------------
KJ361502.1       tgtacttttccc------------------------------------------------
                 .    .* * .

MN908947.3       tggtgttgtttgtacagaaattgaccctaagttggacaattattataagaaagacaattc
NC_006577.2      ------------cttaaaatttaaccc---------------------------agactt
NC_006213.1      ------------cttaaaatttgaaac---------------------------aaactt
NC_002645.1      ---------cactacagtattcgaaac---------------------------cccatt
NC_005831.2      ---------ttttacagtatcttaagc---------------------------ctactt
KJ361502.1       ---------cggccaaaaatacagtag---------------------------cgattg
                             .  *. *   . .                              *

MN908947.3       ttatttcacagagcaaccaattgatcttgtaccaaaccaaccatatccaaacgcaagctt
NC_006577.2      ttacatc--------------------tatgttgac------------------------
NC_006213.1      ttaaatc--------------------ggtgttaac------------------------
NC_002645.1      ttatttcacaaggtc-----ttgatgctgcgtggaa------------------------
NC_005831.2      ttaaatctaagggtt-----taaatgttctttggaa------------------------
KJ361502.1       taatgtcgtacggta-----------ttctttggac------------------------
                 * *  **                      . . .*

MN908947.3       cgataattttaagtttgtatgtgataatatcaaatttgctgatg-atttaaaccagtta-
NC_006577.2      ----------taattatttttggatgatgttgaaatggttgct--tataaccctgatctt
NC_006213.1      ----------cacctattatttggatgatgttaagaaaattgag--tataaacctgacttg
NC_002645.1      ----------taaatttgttttaggcgatgttgaaatttttgttg-catttgtttactatg
NC_005831.2      ----------caaatttgttacaggtgatgttggaccttttgtta-gttttatttatttta
KJ361502.1       --------------------------ggtaatttcagaacagaggttgatcccgacctatctgc
                                           *...** *....       *       .    ... .

MN908947.3       cagtcgctacaggattggcaactataaattaaacacagaccattccagtagcagtgacaa
NC_006577.2      tgttaagtctaaagttggtaactatcg---------------------------------
NC_006213.1      tgttaagtccaaagtcggtaattaccg---------------------------------
NC_002645.1      cgtacgagtaaaacacggtgattttt----------------------------------
NC_005831.2      cgtccgtgctaaacatggtgattttt----------------------------------
KJ361502.1       ccatagatataaggcaggtaattacagg------------------------agtccgcc
                 .    .     *..    **..*.* .

MN908947.3       tattgctttgcttgtacagtaagtgacaacagatgtttcatctcgttgactttcaggtta
NC_006577.2      -tttaccgtct---------------------------------agtaaacctag-----
NC_006213.1      -actgccatc--------------------------------------aacccaaaaggg
NC_002645.1      --ctgcagtg------------------------------------------------ag
NC_005831.2      --ctggtgtt------------------------------------------------gc
KJ361502.1       tattacggcggat-------------------------------attgaacttgcattgc
                   .*.    .

MN908947.3       ctatagcagagatattactaattattatgaggacttttaaagtttccatttggaatcttg
NC_006577.2      ---tggtatggatactgc--cttgttaagag------------------cttaaatctaa
NC_006213.1      ttctggcatggacaccgc--attgttgagaa------------------ataatatctaa
NC_002645.1      ctctcccatgagcaacat--gacagaaaacga------aagattgcttcatttttctaa
NC_005831.2      ctctcaggagggtgtttt--gtcagaaagaga------gaagttgcttcatttaatctaa
KJ361502.1       ttcgagcttaggctcttt-----agtaagagt-----------------atcttaattga
                          ....  . .     .  . . .                 *      .* .

MN908947.3       attacatcataaacctcataattaaaaatttatctaagtcactaactgagaataaatatt
NC_006577.2      actattagg--------------------------atgtcttatactcccggtcattatg
NC_006213.1      attttaagg--------------------------atgtcttttactcctggtaagcaat
NC_002645.1      actgaacg----------------------------------------------------
NC_005831.2      actaaac-----------------------------------------------------
KJ361502.1       ttttaacga---------------------------------------------------
                  .*
```

```
MN908947.3      attacatcataaacctcataattaaaatttatctaagtcactaactgagaataaatatt
NC_006577.2     actattagg--------------------------atgtcttatactcccggtcattatg
NC_006213.1     attttaagg--------------------------atgtcttttactcctggtaagcaat
NC_002645.1     actgaacg----------------------------------------------------
NC_005831.2     actaaac-----------------------------------------------------
KJ361502.1      ttttaacga---------------------------------------------------
                 .*

MN908947.3      ctcaattagatgaagagcaaccaatggagattgattaaacgaacatgaaaattattcttt
NC_006577.2     c-----tggaagtagaagctcctctggaaatcgttc------------------------
NC_006213.1     c-----cagtagtagagcgtcctctggaaatcgttctggta-------------------
NC_002645.1     -----------aaaagatggctacagtcaaatgggctgatgcatctgaa-----------
NC_005831.2     -------------aaaatggctagtgtaaattgggccgatg-------------------
KJ361502.1      ------------------------------------------------------------

MN908947.3      tcttggcactgataacactcgctacttgtgagctttatcactaccaagagtgtgttagag
NC_006577.2     ------------------------------------------------------------
NC_006213.1     ------------------------------------------------------------
NC_002645.1     ------------------------------------------------------------
NC_005831.2     ------------------------------------------------------------
KJ361502.1      -----------------------------------------------------------a

MN908947.3      gtacaacagtacttttaaaagaaccttgctcttctggaacatacgagggcaattcaccat
NC_006577.2     ------aggaatcctcaagaaaacttc---------------------------------
NC_006213.1     -----atggcatcctcaagtggccga----------------------------------
NC_002645.1     ccacaacgtggtcgtcagggtagaat----------------------------------
NC_005831.2     -----acagagctgctaggaagaaatt---------------------------------
KJ361502.1      tctcaatttcattgttatggcagcccc------------------------------tgc
                            ... ..* .  ..

MN908947.3      ttcatcctctagctgataacaaatttgcactgacttgctttagcactcaatttgcttttg
NC_006577.2     ------------------------ttgggctgaccaatctgag-----------------
NC_006213.1     ------------------------tcagtccgaccagttta-------------------
NC_002645.1     ---------------------------accttattctcttt-------------------
NC_005831.2     ------------------------tcctcctccttcatttt-------------------
KJ361502.1      tgcacc------------------tcgtgctgtttcctttg-------------------
                                            *.  ..  ..*

MN908947.3      cttgtcctgacggcgtaaaacacgtctatcagttacgtgccagatcagtttcacctaaac
NC_006577.2     ------------------------------------------------------------
NC_006213.1     ------------------------------------------------------------
NC_002645.1     ------------------------------------------------------------
NC_005831.2     ------------------------------------------------------------
KJ361502.1      ------------------------------------------------------------

MN908947.3      tgttcatcagacaagaggaagttcaagaactttactctccaatttttcttattgttgcgg
NC_006577.2     ------------------------------------------------------------
NC_006213.1     ------------------------------------------------------------
NC_002645.1     ------------------------------------------------------------
NC_005831.2     ------------------------------------------------------------
KJ361502.1      --------------------------------------ccgat-----------------

MN908947.3      caatagtgtttataacactttgcttcacactcaaaagaaagacagaatgattgaactttc
NC_006577.2     ------------------------------------------cgaaattaccaaaccttt
NC_006213.1     -------------------------------------------gaaatgttcaaacc---
NC_002645.1     ------------------------------------------------------------
NC_005831.2     ------------------------------------------------------------
KJ361502.1      ------------------------------------------aacaatgatataa-----

MN908947.3      attaattgacttctatttgtgctttttagcctttctgctattccttgttttaattatgct
NC_006577.2     a-----------------------------------------------------------
NC_006213.1     ------------------------------------------------------------
NC_002645.1     ------------------------------------------------------------
NC_005831.2     ------------------------------------------------------------
KJ361502.1      ------------------------------------------------------------
```

Table S1. Results of biosensor-based home testing by volunteers compared with RT-qPCR analysis. This table displays a sample of results obtained from 60 volunteers, showing the blank capacitance values, the measured capacitance values after saliva testing, and the corresponding RT-qPCR result. The table highlights the accuracy of the biosensor in identifying positive and negative SARS-CoV-2 cases.

No	Blank capacity (nF)	Saliva sample capacity (nF)	RT-qPCR result	No	Blank capacity (nF)	Saliva sample capacity (nF)	RT-qPCR result
1	820	789	Negative	31	813	829	Negative
2	790	764	Negative	32	791	781	Negative
3	810	476	Positive	33	834	497	Positive
4	801	813	Negative	34	813	862	Negative
5	910	793	Negative	35	789	811	Negative
6	834	763	Negative	36	901	799	Negative
7	850	805	Negative	37	881	769	Negative
8	798	539	Positive	38	845	781	Negative
9	786	961	Negative	39	859	770	Negative
10	926	776	Negative	40	889	952	Negative
11	750	757	Negative	41	855	924	Negative
12	871	872	Negative	42	989	578	Positive
13	853	736	Negative	43	966	758	Negative
14	759	887	Negative	44	767	780	Negative
15	913	964	Negative	45	857	813	Negative
16	896	828	Negative	46	842	991	Negative
17	968	845	Negative	47	928	946	Negative
18	800	924	Negative	48	972	997	Negative
19	825	815	Negative	49	941	866	Negative
20	923	811	Negative	50	986	911	Negative
21	798	761	Negative	51	884	969	Negative
22	788	801	Negative	52	949	831	Negative
23	805	844	Negative	53	778	785	Negative
24	949	548	Positive	54	803	790	Negative
25	874	708	Positive	55	980	771	Negative
26	773	381	Positive	56	821	520	Positive
27	906	891	Negative	57	772	628	Positive
28	767	785	Negative	58	899	617	Positive
29	886	996	Negative	59	973	695	Positive
30	957	869	Negative	60	994	515	Positive

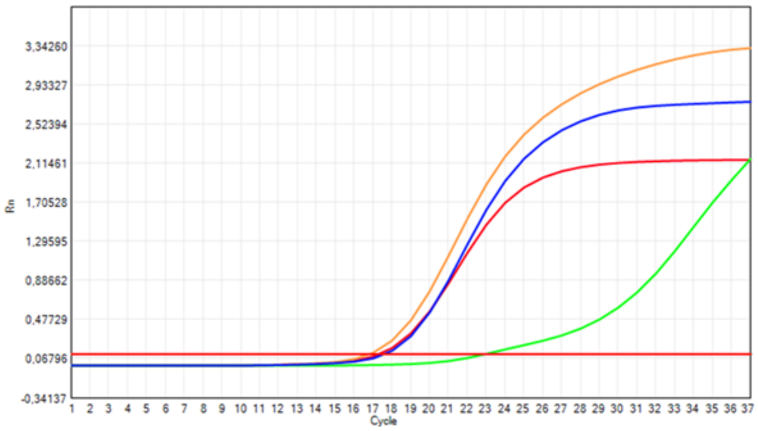

Figure S1. RT-qPCR representative amplification curves for SARS-CoV-2 genes. This figure illustrates the RT-qPCR amplification curves for the ORF1ab, N, and E genes, along with an internal control. The color-coded lines (blue for ORF1ab, green for internal control, orange for gene N, and red for gene E) demonstrate typical amplification patterns and highlight the viral load detected in the samples. This serves as a comparison for validating the biosensor’s results.

Table S2. Biosensor capacitance values for different concentrations of SARS-CoV-2 RNA measured with 10 different electrodes. This table summarizes the capacitance measurements from the biosensor for a range of RNA concentrations across 10 electrodes. It presents the mean capacitance values and standard deviations for each concentration, indicating reproducibility and the consistency of the biosensor’s performance.

Copies	Average capacity [nF]	1	2	3	4	5	6	7	8	9	10
0	860±36	845	920	798	867	855	901	834	879	788	911
10^1	775±27	801	756	780	771	736	759	801	729	759	853
10^2	663±28	650	589	645	689	690	677	650	641	699	701
10^3	558±17	541	566	576	543	571	548	550	569	591	521
10^4	508±12	510	508	514	487	491	500	503	544	499	523
10^5	451±15	461	444	453	471	457	401	453	432	475	462
10^6	408±18	441	445	381	421	399	399	375	411	410	399
